# PrioriTree: a utility for improving phylodynamic analyses in BEAST

**DOI:** 10.1093/bioinformatics/btac849

**Published:** 2023-01-02

**Authors:** Jiansi Gao, Michael R May, Bruce Rannala, Brian R Moore

**Affiliations:** Department of Evolution and Ecology, University of California, Davis, Davis, CA 95616, USA; Department of Evolution and Ecology, University of California, Davis, Davis, CA 95616, USA; Department of Integrative Biology, University of California, Berkeley, Berkeley, CA 94720, USA; Department of Evolution and Ecology, University of California, Davis, Davis, CA 95616, USA; Department of Evolution and Ecology, University of California, Davis, Davis, CA 95616, USA

## Abstract

**Summary:**

Phylodynamic methods are central to studies of the geographic and demographic history of disease outbreaks. Inference under discrete-geographic phylodynamic models—which involve many parameters that must be inferred from minimal information—is inherently sensitive to our prior beliefs about the model parameters. We present an interactive utility, PrioriTree, to help researchers identify and accommodate prior sensitivity in discrete-geographic inferences. Specifically, PrioriTree provides a suite of functions to generate input files for—and summarize output from—BEAST analyses for performing robust Bayesian inference, data-cloning analyses and assessing the relative and absolute fit of candidate discrete-geographic (prior) models to empirical datasets.

**Availability and implementation:**

PrioriTree is distributed as an R package available at https://github.com/jsigao/prioritree, with a comprehensive user manual provided at https://bookdown.org/jsigao/prioritree_manual/.

## 1 Introduction

Phylogenies are increasingly used to study the dispersal history and dynamics of pathogens. The phylodynamic methods developed by Lemey *et al.* (2009) and Edwards *et al.* (2011) are frequently used to infer key aspects of the geographic history of disease outbreaks, including: (1) the area in which an epidemic originated; (2) the dispersal routes by which the pathogen spread among geographic areas, and; (3) the number of dispersal events between areas.

The process of geographic dispersal among a set of discrete areas is modeled as a continuous-time Markov chain. For a geographic history with k areas, this stochastic process is fully specified by a k×k instantaneous-rate matrix, **Q**, where an element of the matrix, *q_ij_*, specifies the instantaneous rate of dispersal from area *i* to area *j*. An additional parameter, *μ*, specifies the average dispersal rate among all areas. We estimate parameters of these phylodynamic models within a Bayesian statistical framework using the Markov chain Monte Carlo (MCMC) algorithms implemented in BEAST (Drummond *et al.*, 2012; Suchard *et al.*, 2018). This approach requires that we first specify a prior probability distribution for each parameter (reflecting our beliefs about that parameter before evaluating the study data); the prior is then updated by the information in the data to return the corresponding posterior probability distribution (reflecting our updated beliefs about the parameter in light of our study data).

To enhance the realism of phylodynamic inferences, empirical studies frequently adopt a granular discretization of continuous geographic space into many discrete areas (Gao *et al.*, 2022a). However, the complexity of geographic models increases rapidly as we increase the number of areas. For example, under an asymmetric geographic model (where the dispersal rate from area *i* to area *j* is allowed to differ from the dispersal rate from area *j* to area *i*), a geographic inference with k=5 areas has 20 pairwise dispersal-rate parameters, with k=10 areas has 90 such parameters and with k=20 areas has 380 such parameters. In every case, we must estimate these parameters from a dataset with minimal information; a single geographic observation (the area where each pathogen was sampled). This inference scenario raises concerns about *prior sensitivity*, *i.e.*, where posterior parameter estimates are strongly influenced by our choice of priors on the model parameters.

We have previously demonstrated that prior sensitivity is, in fact, a pervasive feature of empirical phylodynamic studies (Gao *et al.*, 2022a). This inherent prior sensitivity is of particular concern because the priors on discrete-geographic model parameters implemented as the defaults in BEAST—and used in the vast majority of phylodynamic studies—reflect extremely strong and biologically unrealistic assumptions about the underlying dispersal process (Gao *et al.*, 2022a). These considerations motivated our development of PrioriTree, an interactive utility for identifying and navigating prior sensitivity in discrete-geographic analyses.

## 2 Features

PrioriTree includes a suite of functions to generate input files for—and summarize output from—BEAST (v. 1.x) analyses that allow users to perform robust Bayesian inference, data-cloning analyses and assess the relative and absolute fit of candidate discrete-geographic (prior) models to empirical datasets. We briefly outline these features below.

### 2.1 Robust Bayesian inference

PrioriTree allows users to assess the prior sensitivity of geographic inferences using a robust Bayesian inference approach (Berger, 1994). This approach simply involves performing a series of MCMC analyses—of the same dataset under the same inference model—where we iteratively change one (or more) priors of our discrete-geographic model for each separate analysis. We then compare the resulting series of marginal posterior probability distributions for a given parameter to assess whether (or how much) our estimates change under different priors. While conceptually simple, the main practical challenge with this approach is deciding which parameters to explore and how many alternative priors to evaluate for each parameter under scrutiny.

PrioriTree allows users to explore a wide range of priors for each of the discrete-geographic model parameters. Moreover, the PrioriTree interface dynamically generates a graphical plot of the specified prior probability distribution to help clarify the biological implications of that prior choice. For example, an investigator may lack intuition (and/or prior knowledge) about the average rate of pathogen dispersal, *μ*; however, PrioriTree provides plots of the prior distribution for the number of dispersal events corresponding to a given choice of prior on *μ*. This may help guide the choice of prior; *e.g.,* we minimally know that a pathogen that occurs in 10 areas must have experienced at least 9 dispersal events. This increased transparency may help researchers identify plausible priors to be explored via robust Bayesian inference.

**Fig. 1.**
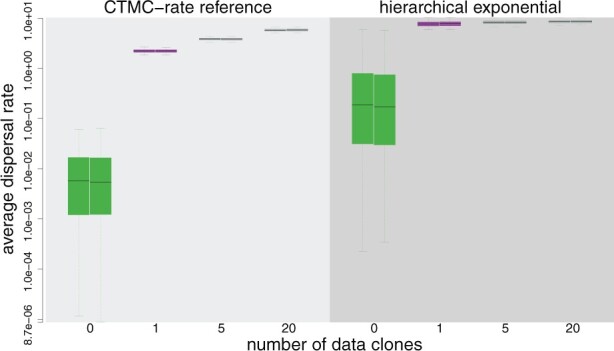
Assessing prior sensitivity in analyses of an example dataset. PrioriTree allows users to assess the prior sensitivity of discrete-geographic analyses performed using BEAST. Here, we explore the prior sensitivity of the average dispersal rate parameter, *μ*. Panels summarize estimates of *μ* under two priors; a CTMC-rate reference prior (used as the default in BEAST, left) and a hierarchical exponential prior (right). Within each panel, each boxplot summarizes the marginal distribution for *μ* inferred for different numbers of data clones (*x*-axis); the prior is inferred without data (green), the posterior is inferred from a single copy (purple) and the data-cloning posteriors are inferred from datasets with 5 or 20 copies (gray). Each pair of boxplots represents replicate analyses (to assess MCMC performance)

PrioriTree provides graphical summaries of prior-sensitivity analyses, plotting distributions for a given parameter under the range of candidate priors (Fig. 1). If the inferred marginal posterior probability distributions for a given parameter are (more or less) identical under a range of corresponding priors, then we can safely conclude that our estimates of this parameter are robust to the choice of prior. Conversely, if the marginal posterior probability distributions vary substantially (and resemble their corresponding marginal prior probability distributions), then we would conclude that this parameter exhibits prior sensitivity.

### 2.2 Data-cloning analyses

PrioriTree also allows us to assess the prior sensitivity of our discrete-geographic inferences using an approach called data cloning (Lele *et al.*, 2007; Ponciano *et al.*, 2009, 2012; Robert, 1993). In contrast to robust Bayesian inference, which explores the impact of *different* prior choices, data cloning is a tool for assessing the impact of a *specific* prior. Intuitively, data cloning measures the relative contribution of the data and the prior to the posterior distribution: an analysis is prior sensitive when the prior makes a relatively large contribution to the posterior. In practice, we perform a series of MCMC analyses—under the same inference model with identical priors—where we iteratively increment the number of copies (“clones”) of our original dataset; increasing the number of clones corresponds to increasing the relative contribution of the data to the posterior. We then explore the resulting series of posterior distributions to assess how our estimates change as the level of information in the data increases (*i.e.*, as we increment the number of data clones).

Data cloning involves a series of MCMC simulations, each with a power $\beta_{i}$, the number of copies of the original dataset. When $\beta_{i}=0$, we are targeting the joint prior probability distribution (*i.e.*, we would be running the MCMC without data), when $\beta_{i}=1$, we are targeting the joint posterior probability distribution (*i.e.*, we would be running the MCMC using our original dataset). As $\beta_{i}\to \infty$, the marginal posterior distribution for the parameter under consideration will converge to a point value that is identical to the maximum-likelihood estimate (MLE) for that parameter (assuming the parameter is identifiable).

Of interest here is the relative rate at which the marginal posterior probability distribution for the parameter under scrutiny—given the prior specified for that parameter—converges to the MLE as we increase the clone number. If the prior is very informative (*i.e.*, focused on a narrow range of parameter values) and/or the prior mean is far from the MLE value, the rate of convergence will be slow. Conversely, if a prior is more diffuse (*i.e.*, spread over a relatively wide range of parameter values) and/or the posterior mean is relatively close to the MLE value, the rate of convergence will be relatively fast.

PrioriTree generates summaries to assess the convergence rate by plotting marginal distributions for a given parameter under the range of $\beta_{i}$ values that were explored. For example, Figure 1 depicts the output from data-cloning analyses of an example dataset (Gao *et al.*, 2022b) to explore the prior sensitivity of the average dispersal rate parameter, *μ*. Under the CTMC-rate reference prior on *μ*, the posterior-mean estimates increase significantly as we increment the number of clones (i.e., where i = 1, 5 or 20 copies of the original dataset; Fig. 1, left panel). Accordingly, this is a highly informative prior on *μ*; i.e., the contribution of this prior to the posterior is large compared to that from the data. By contrast, under the hierarchical-exponential prior on *μ*, the posterior-mean estimates are effectively identical across clone numbers, indicating that this prior is relatively uninformative (Fig. 1, right panel).

### 2.3 Assessing the absolute and relative fit of (prior) models

**Fig. 2.**
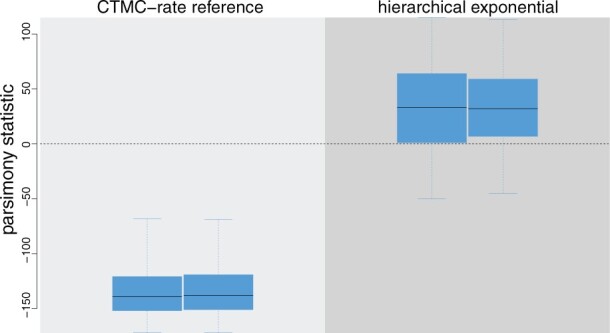
Assessing model adequacy in analyses of an example dataset. PrioriTree allows users to assess the absolute fit of alternative prior models to the data using posterior-predictive simulation. Each boxplot depicts the posterior-predictive distribution of the summary statistic computed from datasets simulated under the CTMC-rate reference prior on *μ* (left panel) or an alternative (hierarchical exponential) prior (right panel). Posterior-predictive distributions under the hierarchical exponential prior overlap with the observed data (dashed horizontal line), indicating that this prior model provides an adequate description of the process that gave rise to the example dataset, whereas the CTMC-rate reference prior is inadequate. Each pair of boxplots represents posterior-predictive summaries for replicate MCMC simulations

PrioriTree implements functions to assess the adequacy (*i.e.*, absolute fit) of the specified (prior) model to our study data using an approach called posterior-predictive simulation (Bollback, 2002; Gelman *et al.*, 1996). This Bayesian approach for assessing model adequacy is based on the following premise: if our inference model provides an adequate description of the process that gave rise to our observed data, then we should be able to use that model to simulate datasets that resemble our original data. The resemblance between the observed and simulated datasets is quantified using a summary statistic. PrioriTree allows users to perform posterior-predictive simulations using the output of BEAST discrete-geographic analyses and then computes and plots the summary statistics to assess model adequacy (Fig. 2). PrioriTree also provides functions to set up power-posterior analyses for estimating the marginal likelihoods of candidate models in BEAST (Baele *et al.*, 2012; Lartillot and Philippe, 2006; Xie *et al.*, 2011) to compare the relative fit of competing (prior) models to the geographic data using Bayes factors (Kass and Raftery, 1995).

### 2.4 Additional features

PrioriTree assumes that the phylogeny and geographic history are inferred sequentially. Under this sequential-inference approach, the phylogeny of the study group is first estimated from a sequence alignment using BEAST. These phylogenetic estimates are then read into PrioriTree as a single summary tree or as a posterior distribution of trees. If the input file contains a posterior distribution of trees, PrioriTree allows users to specify how to marginalize over the distribution to accommodate phylogenetic uncertainty in the discrete-geographic inference.

Users can also set up other BEAST discrete-geographic inferences (*e.g.*, inferring the number of dispersal events between each pair of geographic areas) in PrioriTree. In addition to generating XML scripts (as input files for BEAST analyses) and figures and tables (summarizing various analysis), PrioriTree also dynamically generates an explicit description of the methods and parameters used for each biogeographic analysis to enhance the reproducibility of phylodynamic studies (Magee *et al.*, 2014).

## Data Availability

PrioriTree is developed and distributed as an R Shiny package (Chang *et al.*, 2021; R Core Team, 2021)—that provides a dynamic, graphical-user interface via a local web browser—hosted on GitHub (https://github.com/jsigao/prioritree), with a comprehensive user manual available at https://bookdown.org/jsigao/prioritree_manual/.
